# Systemic Treatment with siRNA Targeting Gamma-Secretase Activating Protein Inhibits Amyloid-β Accumulation in Alzheimer’s Disease

**DOI:** 10.34133/bmr.0027

**Published:** 2024-06-12

**Authors:** Sunghwa Kim, Irfan Ullah, Jagadish Beloor, Kunho Chung, Jongkil Kim, Yujong Yi, Eunhwa Kang, Gyeongju Yun, Seoyoun Heo, Seon-Hong Pyun, Seung Hyun Kim, Priti Kumar, Sang-Kyung Lee

**Affiliations:** ^1^ Department of Bioengineering and Institute of Nanoscience and Technology, Hanyang University, Seoul, Korea.; ^2^ Department of Internal Medicine, Yale University, New Haven, CT, USA.; ^3^ Lerner Research Institute, Cleveland Clinic, Cleveland, OH, USA.; ^4^ Harvard Medical School, Boston, MA, USA.; ^5^ Department of Neurology, College of Medicine, Hanyang University, Seoul, Korea.

## Abstract

Amyloid-β (Aβ) peptide aggregation in the brain is a key factor in Alzheimer’s disease. However, direct inhibition of β-secretase or γ-secretase proves ineffective in reducing Aβ accumulation and improving cognition in Alzheimer’s. Recent findings suggest that inhibiting gamma-secretase activating protein (GSAP) can decrease Aβ generation without affecting crucial γ-secretase substrates. Dimerization of Lep9R3LC (diLep9R3LC) was confirmed by Ellman’s test. The peptide–small interfering RNA (siRNA) complex ratio, particle size, and surface charge were analyzed using electrophoretic mobility shift assay, and dynamic light scattering, respectively. In a 3xTg mice model of Alzheimer’s disease, diLep9R3LC:siRNA complexes were intravenously administered twice a week for 8 weeks. Assessments included gene silencing, protein expression, and behavioral improvement using reverse transcription polymerase chain reaction, quantitative polymerase chain reaction, western blotting, Y-maze, and object recognition tests. The efficacy of Lep9R3LC dimerization was ~80% after a 3-d reaction by Ellman’s test. In N2a cells, diLep9R3LC:siGSAP complexes achieved ~70% silencing at 48 h posttransfection. In 7-month-old male 3xTg mice, GSAP knockdown was ~30% in the cortex and ~50% in the hippocampus. The behavior improved in mice treated with diLep9R3LC:siGSAP complexes, showing a 60% increase in entries and an 80% increase object recognition. A novel dipeptide, diLep9R3LC, complexed with siRNA targeting GSAP (siGSAP), efficiently delivers siRNA to the mouse brain, targeting the hippocampus. The treatment inhibits Aβ accumulation, reduces GSK-3β-associated with tau hyperphosphorylation, and improves Alzheimer’s behavior. Our findings highlight diLep9R3LC:siGSAP’s potential for Alzheimer’s and as a siRNA carrier for central nervous system-related diseases.

## Introduction

Alzheimer’s disease (AD) is prevalent form of dementia among the elderly population, characterized by the formation of amyloid-β (Aβ) plaque, wrinkly brain tissue, loss of volume, and tangled tau deposition in brain cells [1,2]. Both Aβ plaque and tangled tau are neurotoxic and hasten the formation of aggregation [3–5]. Aβ peptide is generated from amyloid precursor protein (APP), cleavage by β-secretase followed by γ-secretase at extracellular domain and transmembrane domain, respectively [3,6–8]. The final length of the Aβ peptides is determined by cleavage position of γ-secretase with high frequency to generate 40- or 42-amino-acids-long sequence of Aβ. These Aβ molecules have high potential to aggregate into oligomers due to its β-sheet-enriched structure, which initiate AD [9–11]. The Aβ oligomers further caused Aβ plaque formation and activation of glycogen synthase kinase-3β (GSK-3β). The exact mechanism of how GSK-3β is activated is unclear; however, the elevated expression of GSK-3β has shown in AD transgenic mice human postmortem brain samples [12,13]. Overexpression of GSK-3β accelerates Aβ production by inducing beta-site APP cleaving enzyme (BACE1) gene expression via upregulation of nuclear factor κB signaling and modulates γ-secretase activity, hyperphosphorylated tau, and neurofibrillary tangles formation in AD condition [13,14]. Many therapeutic strategies were applied to directly inhibit β-secretase or γ-secretase, but unfortunately, this leads to liver toxicity and neuronal abnormalities or inhibition of notch signaling, respectively [15–17]. Thus, the finding of alternative target to inhibit the activity of β-secretase or γ-secretase remains a central goal. Recent studies have shown that gamma-secretase activating protein (GSAP) binds to presenilin-1 (PS1) subunit of γ-secretase and activates the enzyme [18,19]. The GSAP knockout mice significantly reduce Aβ load sparing other γ-secretase substrates especially notch. Further the inhibition of GSAP suppresses Aβ production by conformational change in PS1 in vitro, highlighting it a potential therapeutic target [16,17,20].

A major challenge to treat the neurodegenerative disorders like AD is the development of delivery tools that can easily cross blood–brain barrier (BBB). The BBB is constructed from specialized endothelial cells designed to minimize the uptake of extracellular substances. Further, these cells have extensive tight junctions, significantly limiting cellular permeability. The limited permeability constitutes a restriction in delivering therapeutic drugs to the brain [21,22]. To enter the brain, molecules need to be lipid-soluble, have a molecular weight of less than 400 Da, and should not serve as substrates for active efflux transporters [23,24]. Essential small hydrophilic molecules for the survival of brain cells utilize transporters on both the luminal and basolateral sides of brain endothelial cells [25]. In contrast, larger or hydrophilic essential molecules such as hormones, transferrin, insulin, and lipoproteins rely on specific receptors that are highly expressed on the luminal side of brain endothelial cells [25,26]. These receptors play a role in the endocytosis and transcytosis of molecules across the BBB. Several strategies have been developed to deliver therapeutic drugs to the brain. Neuronal-cell-specific ligand/receptor-mediated endocytosis is an alternative possible strategy to cross BBB [27–29]. We and others used rabbis virus glycoprotein, which binds to acetylcholine receptor expressed on neuronal cells in brain as a delivery moiety to deliver small interfering RNA (siRNA) or exosomes for the central nervous system [29–32]. Other studies also showed successful delivery of DNA to the brain with TAT-modified cholesterol as a form of liposome or antibodies such as transferrin antibody or insulin antibody that binds to its cognate receptor expressed on neuronal cells to deliver antibody-conjugated drugs [33–35]. Another intriguing molecule that is used as a delivery tool to the brain is leptin. Leptin is secreted from adipocytes and enters several parts of the brain via leptin-receptor-expressing cells located throughout the brain [36,37]. A leptin-derived 30-amino-acid peptide conjugated with PEGylated polylysine was confirmed to specifically deliver the plasmid DNA to the brain with similar brain accumulation efficiencies to native endogenic leptin polypeptides after systemic injection [27,38].

In this study, we harnessed the 30-amino-acid leptin-derived peptide that binds to the leptin receptor expressed on brain to deliver small interfering RNA (siRNA) to the brain. Nona-arginine-tri-leucine (9R3LC) with cysteine at the C-terminal end of leptin peptide (Lep9R3LC) was introduced for enhanced siRNA complexation and cytosolic release [29,39]. The leptin-receptors-mediated transport of Lep9R3LC:siRNA complex in the brain provides the opportunity to use leptin peptide as a carrier of siRNA. Further the dimerization of Lep9R3LC (diLep9R3LC) for enhanced binding to its cognate receptor was achieved. With this novel delivery system, we found that the dimer structure of Lep9R3LC delivers siRNA very effectively in various regions of the brain and induced effective gene silencing. Using diLep9R3LC, we confirmed that systemic delivery of siRNA targeting GSAP (siGSAP) effectively controls GSAP expression and inhibits Aβ production, reduces GSK-3, and ameliorates AD-related behavior in AD mice model.

## Materials and Methods

### Peptides

Peptides were synthesized from Peptron (Daejeon, Korea), and sequences are depicted below.

Leptin9R: (​YQQ​VLT​SLP​SQN​VLQ​IAN​DLE​NLR​DLL​HLL​GGG​GRR​RRRRRRRLLLC),

scrambled9R: (EVGQSDYSSYARTSIRASLTFGGGGRRRRRRRRRLLLC).

Three leucines were introduced at the C-terminus for enhanced siRNA release as we have published previously [40]. Peptides were dissolved in phosphate-buffered saline (PBS) containing 30% dimethyl sulfoxide (DMSO) at 100 mg/ml. To make leptin dimer (diLep9R3LC), peptides were incubated for 3 d at room temperature in PBS containing 10% DMSO at 10 mg/ml. Dimerization was confirmed by Ellman’s test using 5,5′-dithiobis-(2-nitrobenzoic acid) as reported previously [39,41,42].

### siRNA

Duplexed siRNA was obtained from ST Pharm (Seoul, Korea). The target siRNA sequence for GSAP was obtained from Dharmacon Inc. (Lafayette, CO, USA) and for CD4 as previously published [29,43]. The siRNA sequences are depicted below.

siRNA targeting GSAP; siGSAP : 5′-AUGCAGAGCUGGACGACAUUU-3′;

siRNA targeting human CD4 [43]; siCD4 : 5′-GAUCAAGAGACUCCUCAGUTT-3′;

Two hundred picomoles of siGSAP was used for in vitro experiments, and 0.3 mg of siRNA/kg was used for in vivo experiments. siRNA targeting CD4 gene (siCD4) was used as a control.

### Size and surface charge measurement

The mean hydrodynamic diameter and ζ-potential of the nanoparticles were measured using dynamic light scattering (Zeta-sizer-nano analyzer ZS; Malvern Instruments, Worcestershire, UK). Particle formation was performed with 100 μg of Lep9R3LC or diLep9R3LC with siRNA at various weight ratios from 1:10 to 1:30. The complexes were performed for 20 min at room temperature in 400 μl of Dulbecco’s PBS (DPBS) (pH 7.4). Size and surface charges were measured 3 independent times.

### Electrophoretic mobility shift assay

Fifty picomoles of siRNA was complexed with different weight ratios (ranging from 1 to 30) of either Lep9R3LC or diLep9R3LC peptide in DPBS (pH 7.4) and incubated at room temperature for 30 min. Samples were loaded onto 1% agarose gels containing ethidium bromide (EtBr) and electrophoresed.

### Biosensor analysis

The direct binding of diLep9R3LC or Lep9R3LC to the target was evaluated using a dual-channel surface plasmon resonance (SPR) instrument (Reichert SR7500DC system, Depew, NY). The process of immobilizing the leptin receptor protein (R&D Systems, Minneapolis, MN) onto the sensor chip surface was accomplished using free amine coupling through an 1-ethyl-3-(3-dimethylaminopropyl) carbodiimide hydrochloride (EDC)/N-hydroxysuccinimide (NHS) reaction. The process of the EDC/NHS reaction involves injecting a mixture containing 0.1 M EDC and 0.05 M NHS. Subsequently, the remaining activated carboxyl groups were quenched with 1 M ethanolamine at pH 6.0. All dilutions of the working peptides were prepared in the running buffer (PBS with 1% DMSO) and injected for 3 min (association time) at a flow rate of 50 μl/min followed by a dissociation phase of 3 min. Nonspecific background binding was subtracted from each sensogram using SPR_V4017 Data Acquisition and Alignment Program (Reichert, Depew, NY). Binding rates and constants were independent of flow rate over a wide range. Best-fit kinetic parameters were obtained by global fitting analysis using Scrubber2 (Biologic Software, Australia).

### Cell culture and transfection

Murine neuroblastoma (Neuro2a, N2a) cells were obtained from American Type Culture Collection (Rockville, MD) and cultured in Dulbecco’s Modified Eagle Medium (DMEM) containing 10% fetal bovine serum, penicillin (100 IU/ml), and streptomycin (100 μg/ml). Peptide:siRNA complexes were at indicated weight ratio in 200 μl of DMEM for 15 min prior to addition to N2a cell in a 12-well plate. Fluorescein isothiocyanate (FITC)-labeled siCD4 was used for cellular uptake assays. To mimic in vitro AD condition, the plasmid-DNA-expressing APP (Addgene, Cambridge, MA) was transfected with Lipofectamine (Invitrogen, Massachusetts, USA) as suggested by the manufacturer.

### Animal experiments

All procedures were performed in compliance with guidelines and protocols approved by the Institutional Animal Care and Use Committee (IACUC) of Hanyang University. The Balb/c mice were used to test the in vivo delivery of complexed particles to the brain. To ascertain ligand-receptor-mediated delivery of diLep9R3LC:siRNA, the leptin-receptor-deficient (db/db, Jackson Laboratory, ME) strains were utilized. Further, to study therapeutic efficacy of siRNA in AD, 7-month-old male 3xTg (B6;129-Psen1tm1Mpm Tg (APPSwe,tauP301L)1Lfa/Mmjax) mice (Jackson Laboratory, ME) were used. These mice show AD-like pathological and physiological characteristics with respect to formation of Aβ plaques and tangled tau as well as abnormal behaviors. For systemic delivery of siRNA, intravenous injections were performed with diLep9R3LC:siRNA complexes (0.3 mg siRNA/kg body weight and 100 μg of diLep9R3LC, 20:1 weight ratio) twice a week for 8 weeks. In all experiments, a group of control mice (*n* = 4) were also injected with DPBS (pH 7.4).

### Analysis of gene silencing

For quantitative polymerase chain reaction (qPCR), total RNA was extracted from N2a cells 24 h posttransfection and homogenized brain samples using the RNAiso kit (Takara, Kyoto, Japan). The RNA was quantified with SYBR premix Ex Taq perfect real time (Takara, Kyoto, Japan) with qPCR primers. In all cases, relative gene expression was calculated in comparison to normalized target mRNA levels in nontreated groups. For reverse transcription PCR (RT-PCR), TaKaRa Taq kit (Takara, Kyoto, Japan) was used with 1 μg of synthesized cDNA with following the primers as suggested by the manufacturer. All primers were synthesized from Cosmogenetech (Seoul, Korea), and sequences are depicted below.

GSAP forward (reverse transcription): TGA TAA CGG AGT GCT GCT GCT TAC TGA

GSAP reverse (reverse transcription): CTG CAC GTC CAC TTT CAT AAG CCC AAA

GSAP forward (quantitative): TGT CCG GCT CCC TCC GCT TAT T

GSAP reverse (quantitative): TTT TTC AGC AGC CGG GCC ACA

GAPDH (glyceraldehyde-3-phosphate dehydrogenase) forward (reverse transcription): ACC ACA GTC CAT GCC ATC AC [44]

GAPDH reverse (reverse transcription): TCC ACC ACC CTG TTG CTG TA [44]

GAPDH forward (quantitative): GGC AAA TTC AAC GGC ACA GT [45]

GAPDH reverse (quantitative): GGG TCT CGC TCC TGG AAG AT [45]

GSK-3β forward (quantitative): AAG CGA TTT AAG AAC CGA GAG C [46]

GSK-3β reverse (quantitative): AGA AAT ACC GCA GTC GGA CTA T [46]

Reverse transcription PCR products were loaded onto 0.7% agarose gels and visualized by ultraviolet light using KODAK MI (Kodak, Stamford, CT).

### Western blot

Proteins were prepared from cell lysates using radioimmunoprecipitation assay buffer as suggested by the manufacturer (Thermo Scientific Pierce, Rockford, IL). Monoclonal antibodies; anti-GSAP (Abcam, Cambridge, England), anti-amyloid-β (Covance, Princeton, NJ), anti-PS1, anti-BACE1 (Millipore, Billerica, MA), β-actin (Santa Cruz Biotechnology, Dallas, TX), horseradish peroxidase-conjugated anti-rabbit immunoglobulin G and anti-mouse immunoglobulin G (Abcam, Cambridge, England) were used. Kodak image station was used to visualize band. Relative band intensities were measured using ImageJ software and normalized to β-actin.

### Thioflavin T assay

Thioflavin T (ThT) solution (200 nM, Sigma-Aldrich, St. Louis, MO) was prepared to detect Aβ fibrils as described previously [47]. Briefly, 20 μg of cell lysate and 50 μl of cell culture medium were incubated with 200 μl of ThT solution, and fluorescence was measured using an ultraviolet/fluorescence reader (spectraMax M2; Molecular devices, Sunnyvale, CA) at an excitation wavelength of 450 nm and emission wavelength of 490 nm. The relative fluorescence intensity was calculated relative to the untreated sample.

### Cytotoxicity assay

Cell viability was analyzed using Cell Counting Kit-8 assay (Dojindo Laboratories, Kumamoto, Japan) at 24 h posttransfection following with the manufacturer`s instructions.

### Thioflavin S staining

Thioflavin S staining was performed according to previously described protocol [48]. Briefly, the paraffin sections were deparaffinized and stained with 0.1% Thioflavin S (Sigma-Aldrich, St. Louis, MO), prepared in 80% ethanol for 15 min. After mounting by 4′,6-diamidino-2-phenylindole-containing mounting medium (Vector, Burlingame, CA), fluorescence images were taken using a confocal microscope (Leica TCS SP5, Wetzlar, Germany) and analyzed using ImageJ software.

### Bio-distribution

Dimeric form of Lep9R3LC (100 μg) was complexed with 400 pmol of siFITC (1:20 weight ratio) and then intravenously injected into mice. After 18 h inoculation, fluorescence image was measured from brain, liver, lung, spleen, kidney, and multiple regions of brain including olfactory bulb, cortex, hippocampus, and thalamus/hypothalamus using image station (Kodak, Stamford, CT).

### Behavioral tests

Behavioral tests were performed following modified protocol based on previously described [49,50]. Briefly, Y-maze test was performed in black top open box (400 mm × 350 mm × 350 mm) containing 3 different objects (similar size but different shape) covered with black paper. Movement of mice were tracked using charge-coupled device camera, and time spending near objects was counted when mice were close to objects by 5 mm. Counting area was set to count only when mouse closed and headed to object. First 30 min was spent to be familiar with stage without objects, and then more 30 min was given to recognize the objects. After 1 h, one of the objects was changed to a new object and tracking were performed. Record of mice who did not stay for more than 5 s for any of objects was excluded.

### Statistical analysis

Statistical analysis of both in vitro and in vivo data used in this study were performed by 2-tailed Student *t* test for assessing differences in mean values between 2 groups and 1-way analysis of variance for assessing differences in mean values among more than 2 groups using GraphPad Prism 5 software. *P* < 0.05 was considered statistically significant.

## Results

### The dimerized-leptin carrier shows enhanced receptor binding affinity and delivery of siRNA

**Fig. 1.**
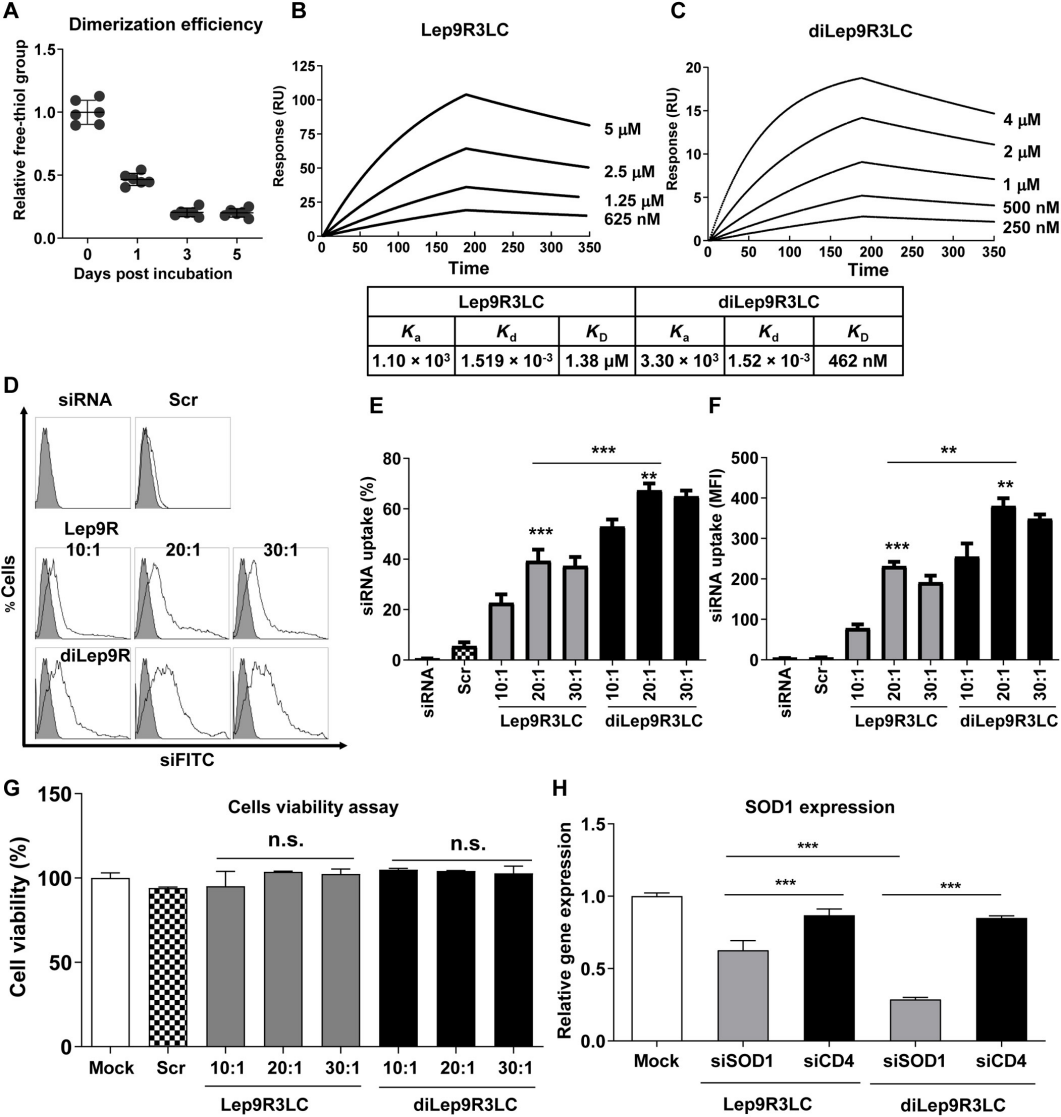
The dimerization of the Leptin9R3LC peptide enhances binding avidity and transfection efficiency. (A) The conjugation efficiency of Lep9R3LC to form diLep9R3LC was accessed by measuring the percentage of relative free thiol groups from cysteine at the C-terminus of peptide using Ellman’s kit. After 3 d of reaction, the conjugation efficiency was determined to be 80% compared to the free cysteine peptide. This measurement was conducted 3 times with a duplex set. (B and C) SPR analysis of diLep9R3LC or Lep9R3LC interaction with leptin receptor. Representative sensograms of SPR showing the interaction of Lep9R3LC (B) or diLep9R3LC (C) at indicated concentrations with a leptin receptor attached on the sensor chip. Rate equations for the association constant (*K*_a_), dissociation constant (*K*_d_), and dissociation constant (*K*_D_) are shown in a box. (D to F) Flow cytometry analysis of N2a cells after an 18-h exposure to Lep9R3LC:siFITC or diLep9R3LC:siFITC at indicated weight ratio. Representative histograms are shown (D) and the cumulative data (E) depicting transfection efficiencies as percent cell and (F) MFIs. Filled or open histogram is correspondence to untransfected or transfected cells with indicated condition, respectively. (G) Cytotoxicity assessment of Lep9R3LC or diLep9R3LC in N2a cells. Cells were treated with Lep9R3LC:siSOD1 or diLep9R3LC:siSOD1 at indicated weight ratios and viabilities were analyzed by Cell Counting Kit-8 assay 24 h posttransfection. (H) qPCR analysis of SOD1 mRNA level in N2a cells treated with Lep9R3LC:siSOD1 and diLep9R3LC:siSOD1 at 20:1 weight ratio 24 h posttransfection. The data represents mean ± SD of 3 independent experiments. siFITC, siRNA labeled with FITC; siCD4, siRNA targeting human CD4; Scr., scrambled peptide. **P < 0.01, ***P < 0.001, n.s., not significant.

We first investigate the physiochemical characterization of leptin-derived peptide. We introduced a cationic nona-argine-3 leucine to Leptin peptide (Lep9R3LC) to complex siRNA and to enhance endosomal escape [40]. The Lep9R3LC was further modified with cysteine residue at terminal peptide to dimerize Lep9R3LC through a disulfide bond to increase siRNA complexation and binding affinity (Fig. S1) as described previously [29]. The efficiency of dimerization of Lep9R3LC was ~80% after 3 d reaction, as measured by Ellman’s test (Fig. 1A). Electrophoretic mobility shift assay shows that diLep9R3LC completely retarded 100 pmol of siRNA at a weight ratio of 20:1, but Lep9R3LC requires a 30:1 weight ratio for complete retardation. This indicates that dimeric form of peptide possesses better siRNA condensation capability (Fig. S2A). Subsequently, we measured the physical and chemical characteristics of Lep9R3LC:siRNA and diLep9R3LC:siRNA nanoparticle for their size and charge at different weight ratio ranges from 10:1 to 30:1. Both diLep9R3LC:siRNA and Lep9R3LC:siRNA forms almost similar nanoparticles, exhibiting a size within the range of 140 to 150 nm and a zeta potential in the range of 32 to 35 mV at 20:1 weight ratio (Fig. S2B and C). This observation indicates that dimerization did not alter siRNA complexation properties and maintain nanoparticles size comparable to those formed by Lep9R3LC. In addition, no differences in the stability of the both peptide:siRNA complexes under various concentrations of heparin (data not shown). Further, to analyze whether diLep9R3LC enhances affinity toward the leptin receptor, we conducted SPR analysis to examine the kinetics of the binding affinity of diLep9R3LC or Lep9R3LC to the leptin receptor. The association (*K*_a_) and dissociation (*K*_d_) constants for diLep9R3LC were determined to be 3.30 × 10^3^ M^-1^S^-1^ and 1.52 × 10^-3^ S^-1^, respectively. For Lep9R3LC, the *K*_a_ and *K*_d_ were estimated at 1.10 × 10^3^ M ^-1^S^-1^ and 1.519 × 10^-3^ S^-1^, respectively. The ultimate binding affinity (*K*_D_) (*K*_d_/*K*_a_) of diLep9R3LC and Lep9R3LC were 462 nM and 1.38 μM, respectively (Fig. 1B and C). The binding affinity (*K*_D_) and dissociation constant (*K*_d_) of diLep9R3LC were significantly better compared to the Lep9R3LC to leptin receptor. To further investigate the delivery capabilities, we tested whether Lep9R3LC or diLep9R3LC exhibits differences in delivering FITC-labeled siRNA (siFITC) into N2a cells. Flow cytometry analysis shows diLep9R3LC is more effective in delivering siRNA than Lep9R3LC (Fig. 1D). The cumulative data from 3 independent experiment clearly indicated that diLep9R3LC delivers ~2 times more siFITC (75%, mean fluorescence intensity [MFI]; 379) compared to that of Lep9R3LC (40.2%, MFI; 288) at 20:1 weight ratio, respectively (Fig. 1E and F). This indicate that diLep9R3LC enhances both the percentage of siRNA uptake and the amount per cell basis. As a control, cells were transfected with siFITC only (2.49%, MFI; 5.15), or scrambled peptide-9R3LC: siFITC (5.19%, MFI; 5.06) at 20:1 weight ratio (Fig. 1D to F). Next, we tested cytotoxicity of Lep9R3LC and diLep9R3LC peptides in N2a cells to ensure that peptides itself are not toxic. Both Lep9R3LC and diLep9R3LC peptides did not show any cytotoxicity at tested weight ratio (Fig. 1G). Since there was no cellular toxicity and enhanced siRNA transfection efficiency at >20:1 peptide:siRNA weight ratio, we restricted at 20:1 weight ratio for further experiment. Next, we assessed the functional delivery of siRNA by these peptides targeting murine superoxide dismutase-1 gene (SOD1). As expected, diLep9R3LC complexed with siSOD1 showed 71% silencing in N2a, while Lep9R3LC:siSOD1 showed 38% of silencing compared to that of control mock (Fig. 1H). Our data clearly indicated that diLep9R3LC had enhanced siRNA complexation and effective silencing with higher delivery of siRNA. Therefore, we stick to used diLep9R3LC as a siRNA carrier for further experiments.

### Inhibition of GSAP reduces Aβ formation in vitro

**Fig. 2.**
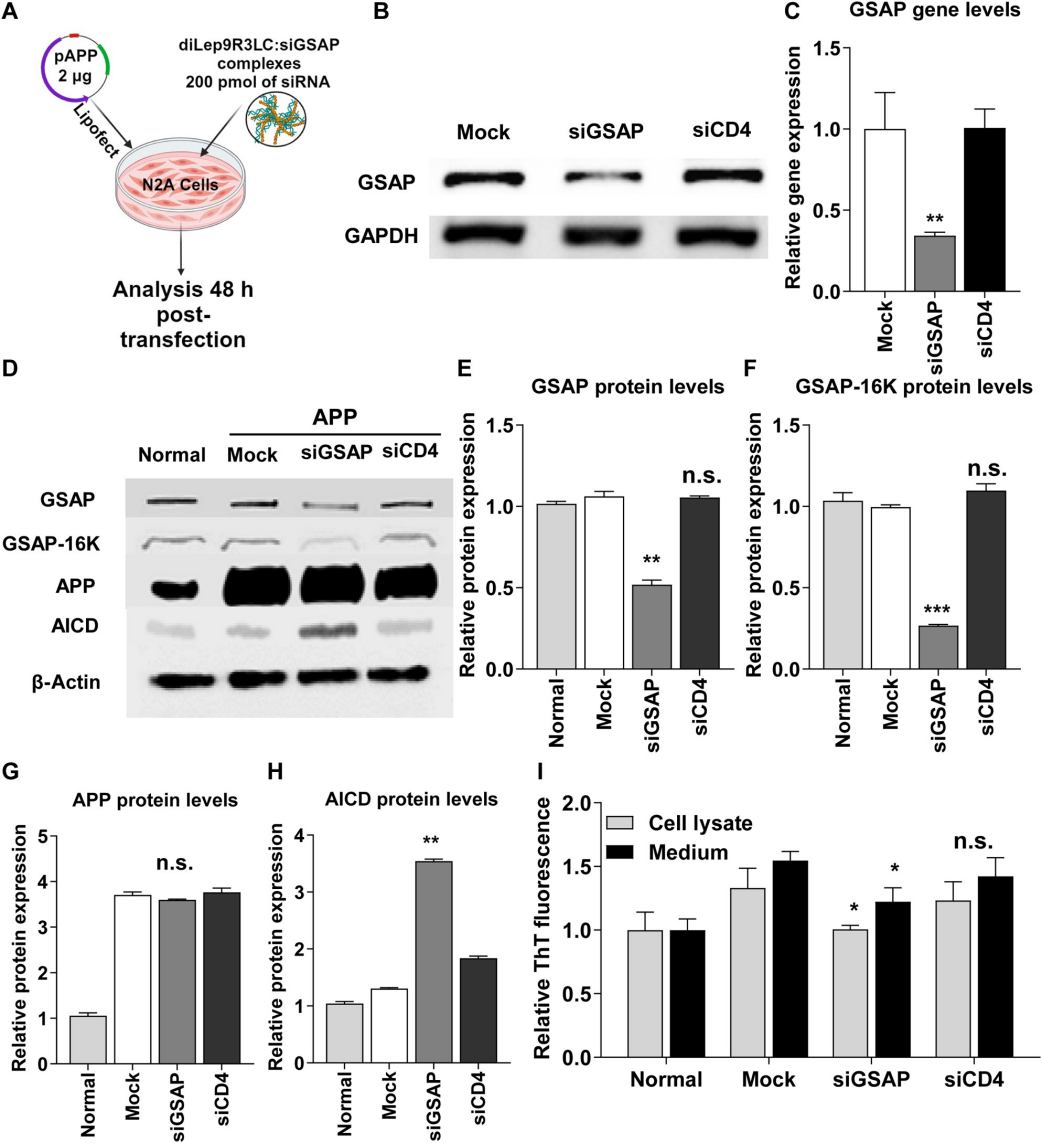
The reduction of GSAP leads to a decrease in Aβ generation in cultured cells. (A) An experimental scheme to test therapeutic efficacy of siGSAP. The N2a cells were cotransfected with plasmid-expressing APP (2 μg, with Lipofectamine) and with diLep9R3LC:siGSAP complexes (200 pmol of siRNA). (B and C) The inhibition of GSAP gene expression by siGSAP was confirmed through both RT-PCR (B) and qPCR (C) analyses in N2a cells. These results were consistent across 3 independent experiments. (D) Representative western blots of indicated protein targets from treated N2a cells. (E to H) Relative levels of protein expression of indicated target proteins normalized with β actin levels. Data represents mean ± SD form 3 independent experiments. (I) The effect of siGSAP on Aβ_42_ levels was confirmed by Thioflavin-T assay after 48 h transfection. Cell lysates (gray bar) and medium (filled bar) were collected, and fluorescence was measured using a spectrophotometer after incubation with ThT. Values were normalized to untreated cells. The data represents the mean ± SD for relative ThT intensity compare to control and untreated mock. *P < 0.05, **P < 0.01, **P < 0.01, ***P < 0.001; n.s, not significant.

It has been proposed that effective inhibition of GSAP can reduce Aβ formation without impairing cleavage of other γ-secretase substrates and tau phosphorylation in a mouse model of Alzheimer’s disease [18]. We first tested effectiveness of siGSAP gene in N2a (Fig. 2A). We found noteworthy decrease (~70% inhibition) of GSAP at 48 h posttransfection with diLep9R3LC:siGSAP by RT-PCR and qPCR (Fig. 2B and C). To evaluate the functional effect of siGSAP on Aβ production, diLep9R3LC:siGSAP was cotransfected with plasmid-expressing APP (pAPP) complexed with Lipofectamine in N2a cells to mimic in vivo disease condition. A representative western blot illustrates that silencing GSAP significantly notably inhibits the C-terminal domain of GSAP (GSAP-16K), an active form of GSAP that bind to γ secretase (Fig. 2D to F). Interestingly, the silencing of GSAP also results in a significant increase in the levels of APP intracellular domain (AICD). This suggests a reduction in abnormal cleavage by γ-secretase complex without affecting the total APP levels (Fig. 2G and H). Moreover, a ThT assay, which utilizes a fluorescent chemical compound binding to β-sheet-rich structures like Aβ, shows 35%, 30% reduction in Aβ production from cell lysate and medium of cells treated with siGSAP, respectively, in comparison to the control cells treated with pAPP only or diLep9R3LC:siCD4 (Fig. 2I). These results indicate that siGSAP inhibits γ-secretase activation by reducing GSAP-16, which leads to exclusive cleavage of APP by β-secretase, leaving transmembrane and cytoplasmic parts of APP resulted in stacking of noncleaved AICD. Thus, our result indicates that inhibiting GSAP reduces activation of γ-secretase result in reduction of neurotoxic Aβ peptides accumulation.

### Dimerized, diLep9R3LC efficiently delivers functional siRNA to the brain

**Fig. 3.**
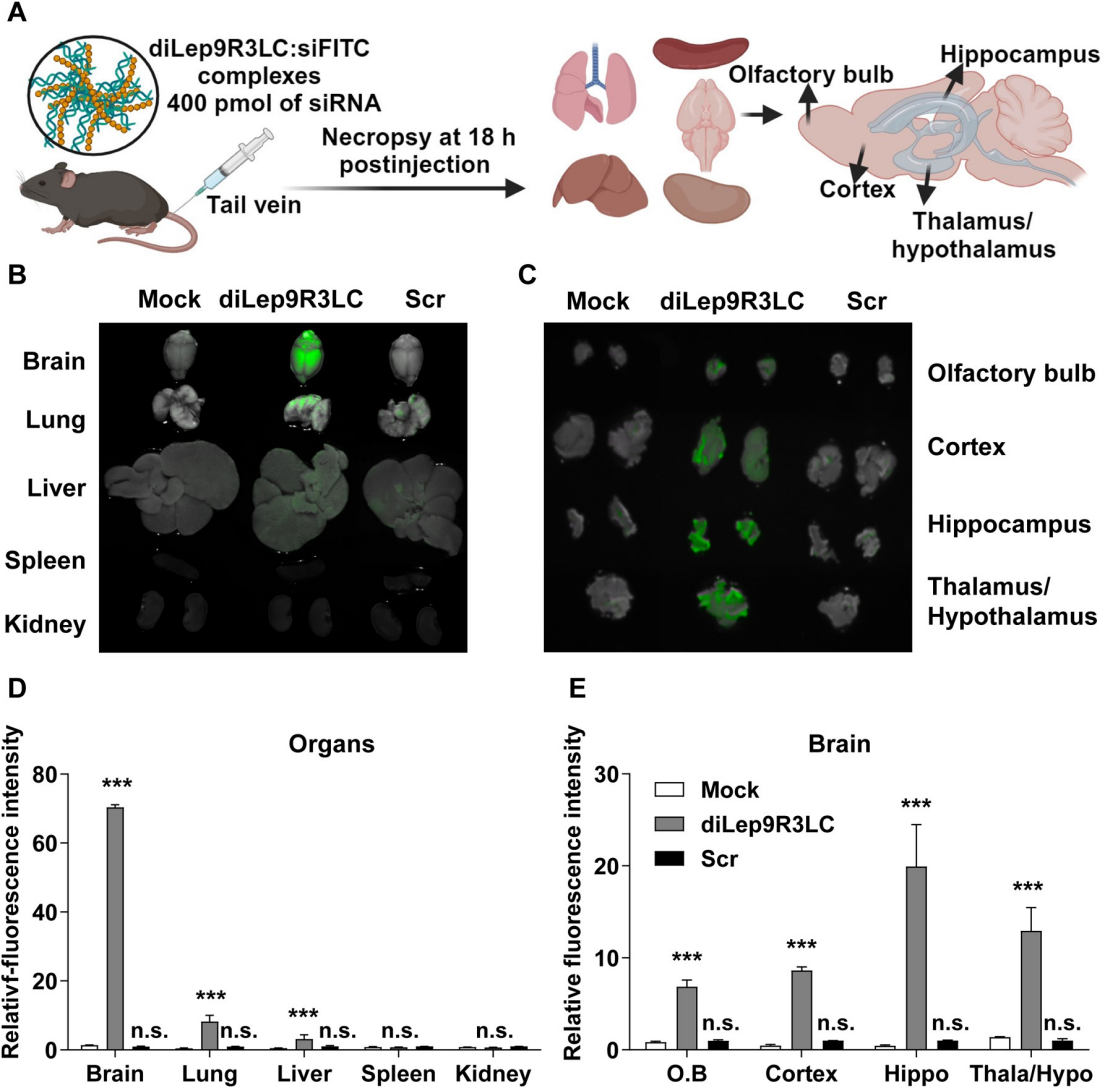
diLep9R3LC facilitates the delivery of siRNA to the brain. (A) An experiment scheme to test siRNA delivery efficiency of diLep9R3LC peptide. The mice were injected with 400 pmol of siFITC siRNA as tracer (tail vein injection), and indicated organs were imaged and quantified for fluorescence after 18 h postadministration. The scrambled peptide was used as control. (B and C) Representative biodistribution of siFITC in brain, lung, liver, spleen, and kidney and dissected brain regions including olfactory bulb, cortex, hippocampus and thalamus/hypothalamus. (D and E) Cumulative data to estimate fluorescent signals from indicated organs from 3 independent experiments (*n* = 8 per group). Results are mean ± SD. Scr, Scrambled peptide; O.B, olfactory bulb; Hippo, hippocampus; Thala/Hypo, thalamus/hypothalamus. **P < 0.01, **P < 0.001; n.s, not significant.

**Fig. 4.**
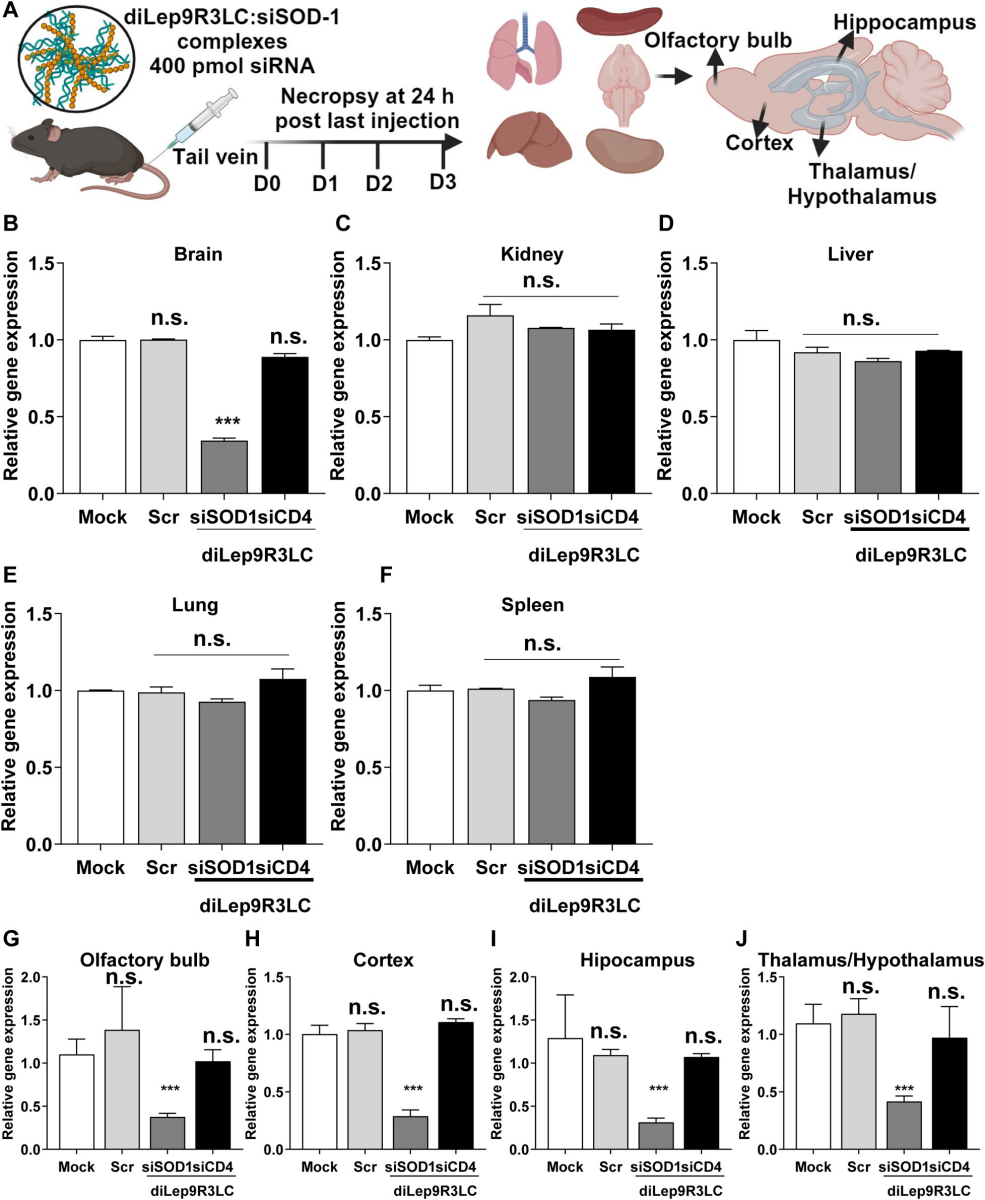
diLep9R3LC has ability to knockdown the activity of target gene in various regions of the brain. (A) An experiment scheme to test functional effect of siRNA using diLep9R3LC peptide. The mice were injected with 400 pmol of siSOD1 siRNA (tail vein injection, daily once for 3 d), and levels of SOD1 gene was quantified in indicated organs. (B to F) Analysis of SOD-1 gene silencing by qPCR in brain, kidney, liver, lung, and spleen after siSOD1 treatment. (G to J) Analysis of SOD1 gene silencing by qPCR in brain regions including olfactory bulb, after siSOD1 treatment. The data is from 3 independent experiments (*n* = 8 per group). The levels of SOD1 gene were normalized to the GAPDH mRNA levels. PBS was used as mock treatment, scrambled peptide was used as a nontargeting peptide, and siCD4 was used as nonspecific siRNA. The data is shown as mean ± SD. Scr, Scrambled peptide; O.B, olfactory bulb; Hippo, hippocampus; Thala/Hypo, thalamus/hypothalamus. **P < 0.01, **P < 0.001; n.s, not significant.

In our subsequent experiment, we assessed the delivery of siRNA into the brain via systemic injection using diLep9R3LC as a carrier. We administered diLep9R3LC:siFITC or scrambled (Scr)9R3LC:siFITC systemically and then monitored fluorescence signals ex vivo from 5 main organs 18 h postinjection (Fig. 3A). Surprisingly, we observed a significant fluorescence signals only in the brain of mice injected with diLep9R3LC:siFITC but not from the scrambled peptide (Fig. 3B and D). No fluorescence signals were detected in the kidney or spleen, but a moderate level of fluorescence was observed in the lung and liver. Upon further examination of dissected brain tissues revealed strong fluorescence signal in hippocampus followed by thalamus/hypothalamus, cortex, and olfactory bulb (Fig. 3C and E). To confirm that specificity of leptin-receptor-mediated siRNA delivery, we delivered siRNA in leptin-receptor-deficient db/db mice (Fig. S3A). Interestingly, systemic delivery of diLep9R3LC:siFITC was not observed in the brain of db/db mice (Fig. S3B and C) despite strong localization in normal mice (Fig. 3). To confirm the functional delivery of siRNA, we systematically administered 400 pmol of siSOD1 or siCD4 complexed with 100 μg of diLep9R3LC or scr9R3LC peptide to mice daily for 3 consecutive days (Fig. 4A). A significant silencing effect was observed in brain only when siRNA was delivered by diLep9R3LC but not when used with scrambled peptide (Fig. 4B). As expected, we did not observe any silencing effect in lung, liver, spleen, or kidney, suggesting that diLep9R3LC enables specific targeting to the brain (Fig. 4C to F). Further in-depth analysis of various brain regions of mice treated with diLep9R3LC:siSOD1 exhibits a substantial reduction in SOD1 expression in olfactory bulb, cortex, hippocampus, and thalamus/hypothalamus by 56%, 70%, 72%, and 60%, respectively (Fig. 4G to J). However, this effect was not observed in the brain of leptin-receptor-deficient (db/db) mice (Fig. S3D to H). Our data clearly demonstrates the systemic delivery of functional siRNA to brain by diLep9R3LC with highest levels of silencing observed in the hippocampus.

### GSAP knockdown improve AD behavior by lowering brain Aβ levels

**Fig. 5.**
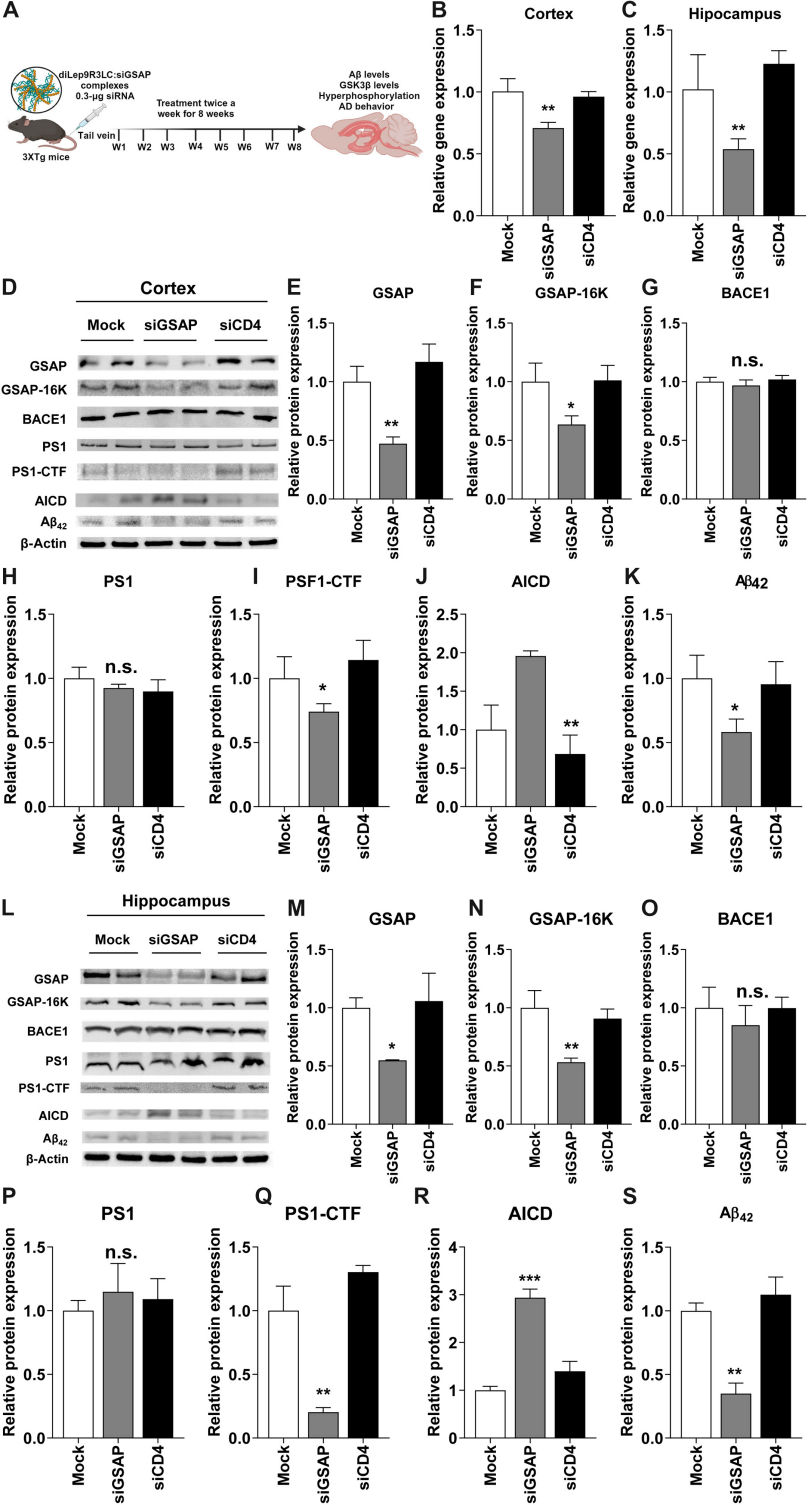
GSAP inhibition reduces Aβ levels in brain of AD mice. (A) An experiment scheme to test functional effect of siRNA using diLep9R3LC peptide in AD mouse model. The mice were injected with 0.3 μg of siGSAP siRNA (tail vein injection, twice a week for 8 wk). (B and C) Analysis of siGSAP gene silencing by qPCR in both cortex and hippocampus (*n* = 4 per group). Control mice treated with siCD4 (*n* = 8 per group) and nontreated mice (*n* = 8 per group) were used as controls. (D) Representative western blot of proteins involved in Aβ generation from cortex. (E to K) Relative levels of protein expression of indicated target proteins normalized with β actin levels in cortex (*n* = 8 per group). (L) Representative western blot of proteins involved in Aβ generation from hippocampus. (M to S) Relative levels of protein expression of indicated target proteins normalized with β actin levels in hippocampus (*n* = 8 per group). The data represents mean ± SD. PBS-treated mice were used as mock control and siRNA-targeting CD4 was used negative control. *P < 0.05, **P < 0.01, ***P < 0.001; n.s, not significant.

**Fig. 6.**
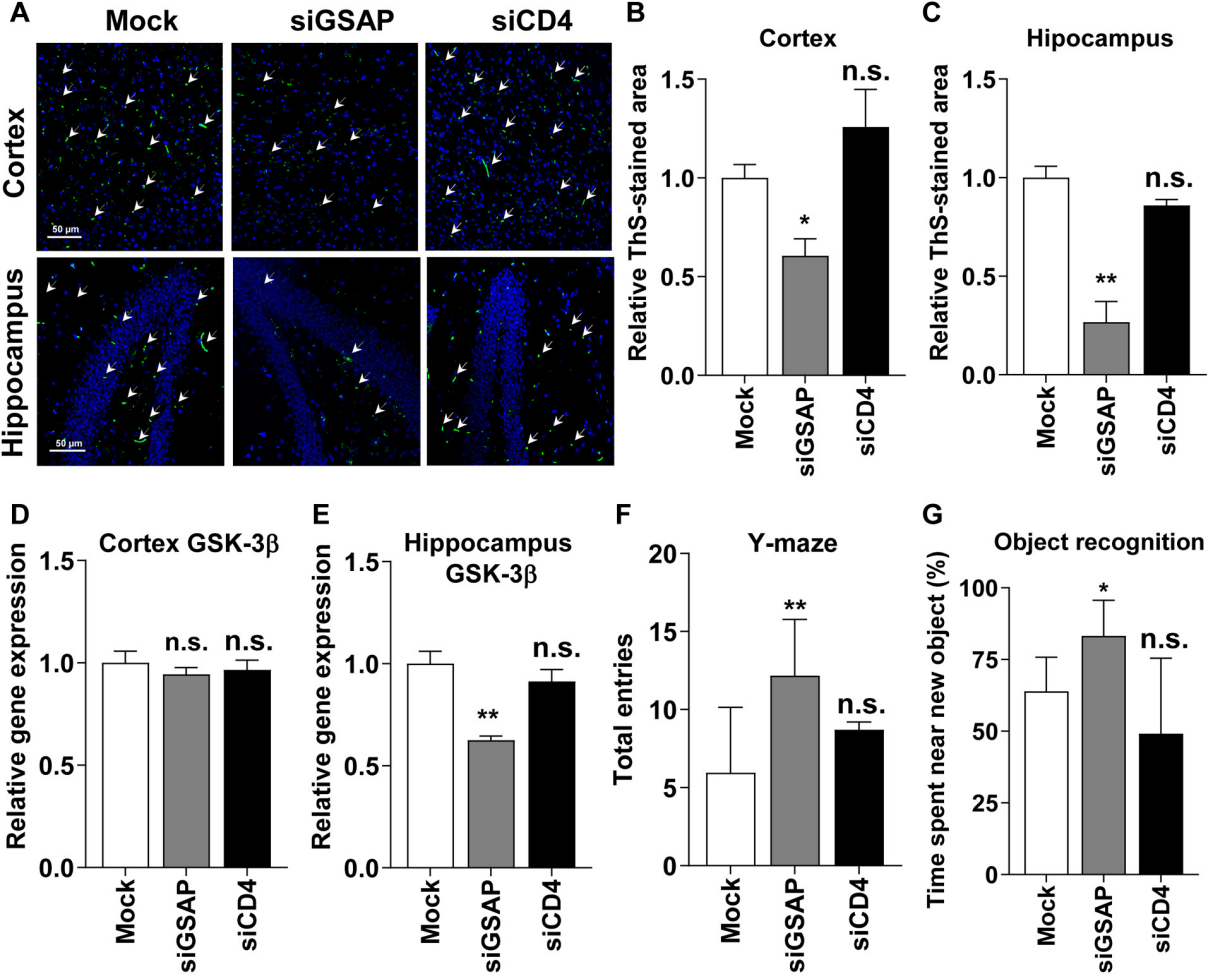
GSAP inhibition reduces Aβ plaques and improve behavior in AD mice. (A) Depicted are representative paraffin-embedded sections stained with Thioflavin-S, which binds to Aβ plaques (shown in green) (*n* = 8 per group). The scale bar represents 50 μm. (B and C) The relative Thioflavin-S-positive stained area serves as a measure for Aβ plaques in the cortex and hippocampus. The relative Thioflavin-S-positive area was normalized to the total pixel number. (D and E) GSK3β expression was assessed by qPCR in the cortex and hippocampus. (F and G) Behavioral changes were estimated through Y-maze and object recognition analyses (*n* = 8 per group). Y-maze data were recorded when all of the mouse’s feet entered each of the ways, and object recognition was measured based on the time spent near the new object. PBS-treated mice were used as a mock control, and siRNA-targeting CD4 was employed as a negative control. The data represent the mean ± SD for 5 individual experiments. *P < 0.05, **P < 0.01, ***P < 0.001; n.s, not significant.

We next evaluate whether systemic administrations of siGSAP by diLep9R3LC that target GSAP can reduce Aβ production and improve AD condition in triple transgenic Alzheimer disease model (3xTg-AD). The 3xTg-AD mice were designed to express 3 key genetic mutations (APP, PS-1, and Tau protein) associated with Alzheimer’s disease, making them a valuable tool for studying the disease’s development and progression [51]. We first verified the gene expression of GSAP in these 3xTg-AD mice model. Our data showed an age-dependent increase in GSAP expression, notably in hippocampus compared to cortex starting from 7-month-old mice (Fig. S4A and B). Therefore, we chose 7-month-old AD mice to test the therapeutic effects of siGSAP on AD condition. The mice were systemically inoculated (0.3-mg siRNA/kg) twice a week with diLep9R3LC:siGSAP for a duration of 8 weeks. Two days after final inoculation, brain tissue was harvested, and GSAP expression was quantified by qPCR in cortex and hippocampus of mice received siGSAP as well as in control mice inoculated with siCD4 or mock treatments (Fig. 5A). In contrast to mock-treated cohort, we observed about ~30% knockdown of GSAP in the cortex and ~50% in the hippocampus (Fig. 5B and C). The treatment with siGSAP led to reduce the protein levels of GSAP above 50% both in cortex and brain (Fig. 5E and M). Subsequently, we quantified the level of proteins implicated in the synthesis of Aβ generation in the cortex as well as in the hippocampus from mice treated with siGSAP, siCD4, or mock (Fig. 5D and L). Silencing GSAP led to a reduction of its active form GSAP-16K by 37% in the cortex and 47% in the hippocampus (Fig. 5F and N). However, the expression of β-secretase (BACE1) and PS1 was not altered by siGSAP as these proteins are not directly linked to γ-secretase (Fig. 5G, H, O, and P). In consistency with a previous study [18], the reduction of GSAP resulted in a 26% inhibition of PS1-CTF in the cortex and 60% in the hippocampus (Fig. 5I and Q). The reduction of GSAP and GSAP-16K significantly elevates AICD by 1-fold in cortex and 2-fold in hippocampus (Fig. 5J and R). Inhibition of GSAP eventually led to a 40% reduction Aβ accumulation in cortex by and hippocampus by 66% (Fig. 5K and S). Additionally, paraffin-embedded brain section from brain samples, stained with Thioflavin S, which binds to Aβ, clearly showed a reduction of Aβ in mice treated with siGSAP when compared to those treated with siCD4 or mock treatment in both the cortex and hippocampus (Fig. 6A). The cumulative data from 3 independent experiments revealed that the reduction of Aβ accumulation was ~40% in the cortex and ~70% in hippocampus from mice that received siGSAP (Fig. 6B and C). We then tested whether the inhibition of GSAP had an impact on GSK-3β expression since its expression and activity can increased due to an accumulation of Aβ in the brain [52,53]. In our AD mouse model, we observed an age-dependent increase in the levels of GSK3β expression in both hippocampus and cortex. (Fig. S5). Intriguingly, we found that treatment with siGSAP inhibited GSK3β expression by ~40%, but this effect was observed only in the hippocampus, not in the cortex (Fig. 6D and E). The observed discrepancy may indeed be due to the moderate level of Aβ deposition in the cortex region affecting in GSK3β expression, as indicated in (Fig. 6A) [54]. Finally, we test whether reduction of GSAP had an impact on the behavior of AD mice since Aβ accumulation was reduced significantly in hippocampus. The mice were subjected to Y-maze and objective recognition tests. As we expected, we found significantly ameliorated behaviors from both Y-maze and object recognition test (Fig. 6F). Mice treated with siGSAP exhibited ~60% increase in entries and ~80% increase in object recognition (Fig. 6G). Thus, our data clearly demonstrate that systemic treatment of siGSAP ameliorates AD by reducing GSAP, inhibiting γ-secretase, reducing GSK3β expression and reducing Aβ accumulation in the brain.

## Discussion

Despite recent advancements in drug delivery to various body organs, targeting the brain specifically remains a significant challenge. The primary obstacle to achieving brain-specific delivery is the presence of BBB. Numerous studies have been conducted to overcome the BBB for the delivery of nucleic acids, proteins, and peptide by attaching ligands that bind to their cognate receptor expressed on endothelial surface of BBB [27,29,55,56]. Harnessing endocytic ligand receptor expressed on brain endothelial cell is a clinically viable approach to delivering drugs to the brain [57]. Among these ligands, a 30-amino-acid leptin-derived peptide has shown specific brain localization after binding to its cognate receptor expressed in all regions of brain [27]. While this ligand approach facilitate binding to receptors on brain endothelia cells to enable drug delivery to the brain, there is a limitation related to poor biding affinity toward the target receptor. This often necessitates high dose or repetitive injection to achieve efficient delivery. Increasing biding affinity of these ligands to receptor expressed on brain cells could potentially enhance the delivery of therapeutic cargos to the brain. A simplest approach is to dimerize the ligands to maximize the avidity toward its receptor. In our study, we first dimerized the leptin peptide to improve avidity, followed by conjugation to 9R3LC (diLep9R3LC) to enable siRNA complex formation and for systemic delivery to the brain. Our results support the notion that dimeric peptide has better avidity over monomeric peptide to its cognate ligand [58]. Our diLep9R3LC peptide enables better siRNA delivery with maximum silencing in in vitro-cultured N2a cells indicating that enhanced binding avidity may influence the delivery of therapeutic cargo (Figs. 1B and C and 2B and C). Further, the systemic administration of diLep9R3LC demonstrated highly efficient delivery of functional siRNA to the brain while avoiding delivery to other organs (Fig. 3). It is worth noting that many studies on systemic siRNA delivery have focused on certain disease conditions like brain tumor, brain hypoxia, or virus infection, which may be more favorable in abnormal BBB, to facilitate brain delivery [59,60]. Thus, evaluating the delivery system effectiveness in reaching the normal brain is an essential step, particularly in the context of AD. A recent application involves the successful delivery of functional siRNA to the normal brain through rabbis virus glycoprotein-modified dendritic-cell-derived exosome [31]. It may need to test whether autologous dendritic cell is required to make exosome-modified brain delivery vehicles. The diLep9R3LC used in in our study allowed enhanced brain-specific delivery of functional siRNA only to the brain without nonspecific delivery to other organs (Fig. 4). It is noteworthy to mention that advantage of using siRNA in treatment of neurological disorders stems from unique characteristics of neuron’s, as they are terminally differentiated cells. We have previously shown that siRNA can induce silencing for a duration of up to 21 d in nondividing cells, such as macrophages [61]. Thus, it is practically feasible to use siRNA as a drug to silence target genes in the brain with minimal need for repeated inoculation. Nevertheless, the toxicity of nona-arginine as a gene carrier and the potential side effects of leptin peptide remain uncertain. Clinical trial NCT00264706 is currently examining the toxicity of nona-arginine. Barrett et. al. [62] reported that 4-week intravenous injection of leptin peptide resulted in weight increases in female rats, with no such effects observed in male rats. Therefore, additional experiments are necessary to explore potential side effects of both nona-arginine and leptin peptide in mice. While our study suggests the overall safety of nona-arginine and leptin peptide, it is crucial to consider potential risks in future clinical trials. Continuous monitoring of these trials will ensure ongoing safety assessments, addressing the need for a comprehensive understanding of nona-arginine toxicity in various clinical contexts.

In addition to the challenge of delivering the genes specifically to the brain, certain neurodegenerative disorders like Alzheimer disease present a complex dilemma when it comes to selecting a promising gene target. Numerous therapeutic targets have been identified, but majority of drug candidates aimed at these targets have not shown efficacy in slowing down the progression of AD [63]. Over the last decade, significant attention has been directed toward interfering with the production of Aβ peptide production, primarily focusing on γ-secretase inhibition due to its critical role in the disease progression. Unfortunately, many therapeutic drugs designed to target γ-secretase have failed in clinical trials, primarily because of their adverse effects on other substrates, especially the notch signaling pathway, in addition to APP [64]. Hence, the selection of therapeutic target in AD while avoiding the adverse effects remains a paramount consideration in treatment strategies. In our current study, we focused on targeting GSAP, a key player in the activation of γ secretase, as a promising candidate for inhibiting the accumulation of Aβ [65]. Our results clearly demonstrated that the inhibition of GSAP through the systemic administration of diLep9R3LC:siGSAP effectively suppresses GSAP levels and reduces Aβ accumulation in hippocampus without altering BACE or PS1 activity (Fig. 5). It is intriguing to note that reduction of Aβ deposition in the brain, particularly in the hippocampus, also has an impact on the expression of GSK-3β, a critical kinase involved in the hyperphosphorylation of tau (Fig. 6E). However, it is not clear the why GSK-3β levels did not change in cortex although expression of Aβ was reduced by ~50% in mice treated with siGSAP. It is plausible that the reaming ~50% levels of Aβ in the cortex could activate GSK-3β to accelerate Aβ plaque formation and subsequently AD pathogenesis; however, this requires further investigation [66,67]. Based on our finding, complete elimination or at least inhibition of Aβ at certain threshold is required to inhibit the GSK-3β induction.

In summary, our study employed a delivery system involving dimerized leptin-derived peptide coupled with 9R3LC (diLep9R3LC) to systemically deliver therapeutic siRNA to the brain. The delivery siGSAP by using this approach efficiently reduced γ-secretase levels, GSK3β activity, and Aβ plaque in the brain leading to amelioration of AD progression. Our finding has provided preclinical evidence that targeting GSAP holds promise as an effective target to treat AD. It is also intriguing to explore the therapeutic effects of siGSAP in elderly mice, going beyond the 7-month-old AD mice used in our experiments. Our experimental design primarily focuses on the early onset of Alzheimer’s disease, considering the expression of GSAP or GSK-3β. Consequently, further experiments are warranted to investigate the role of GSAP in the late stages of Alzheimer’s disease, as well as the long-term effects of siGSAP on neuronal cell regeneration in the brain.

## Ethical Approval

All experimental procedures were approved by the IACUC of Hanyang University (HY-IACUC-2013-0111).

## Data Availability

The datasets used and/or analyzed during the current study available from the corresponding author on reasonable request.
