# Supplementary figures and images for: Systemic Treatment with siRNA Targeting Gamma-Secretase Activating Protein Inhibits Amyloid-β Accumulation in Alzheimer’s Disease
Source: Biomater Res. 2024 Jun 12;28:0027. doi: 10.34133/bmr.0027 (PMC11168191; doi:10.34133/bmr.0027)

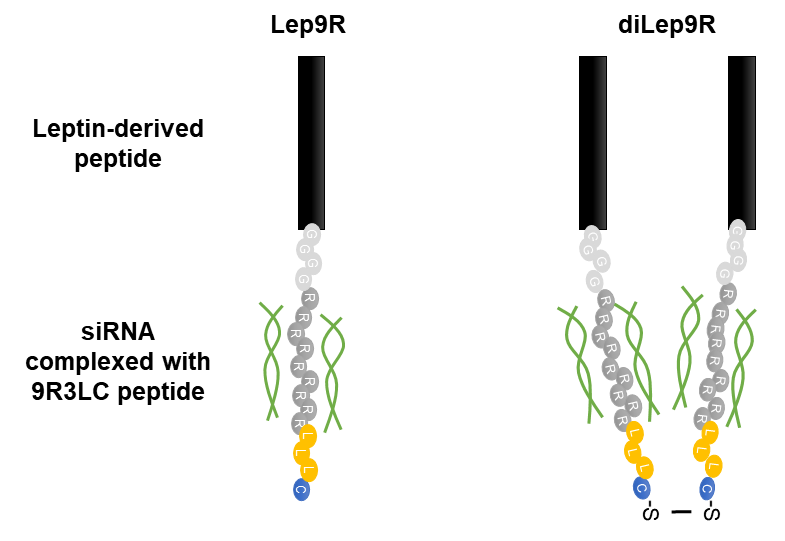

Supplement: Supplementary 1 — Figs. S1 to S5 [file bmr.0027.f1.zip › Fig. S1.png]

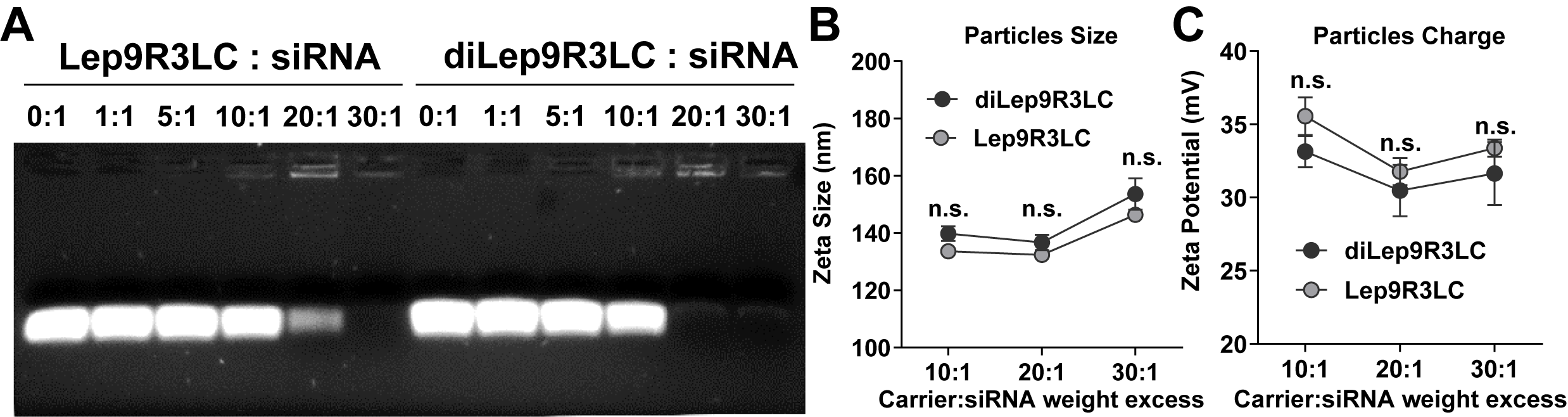

Supplement: Supplementary 1 — Figs. S1 to S5 [file bmr.0027.f1.zip › Fig S2.tif]

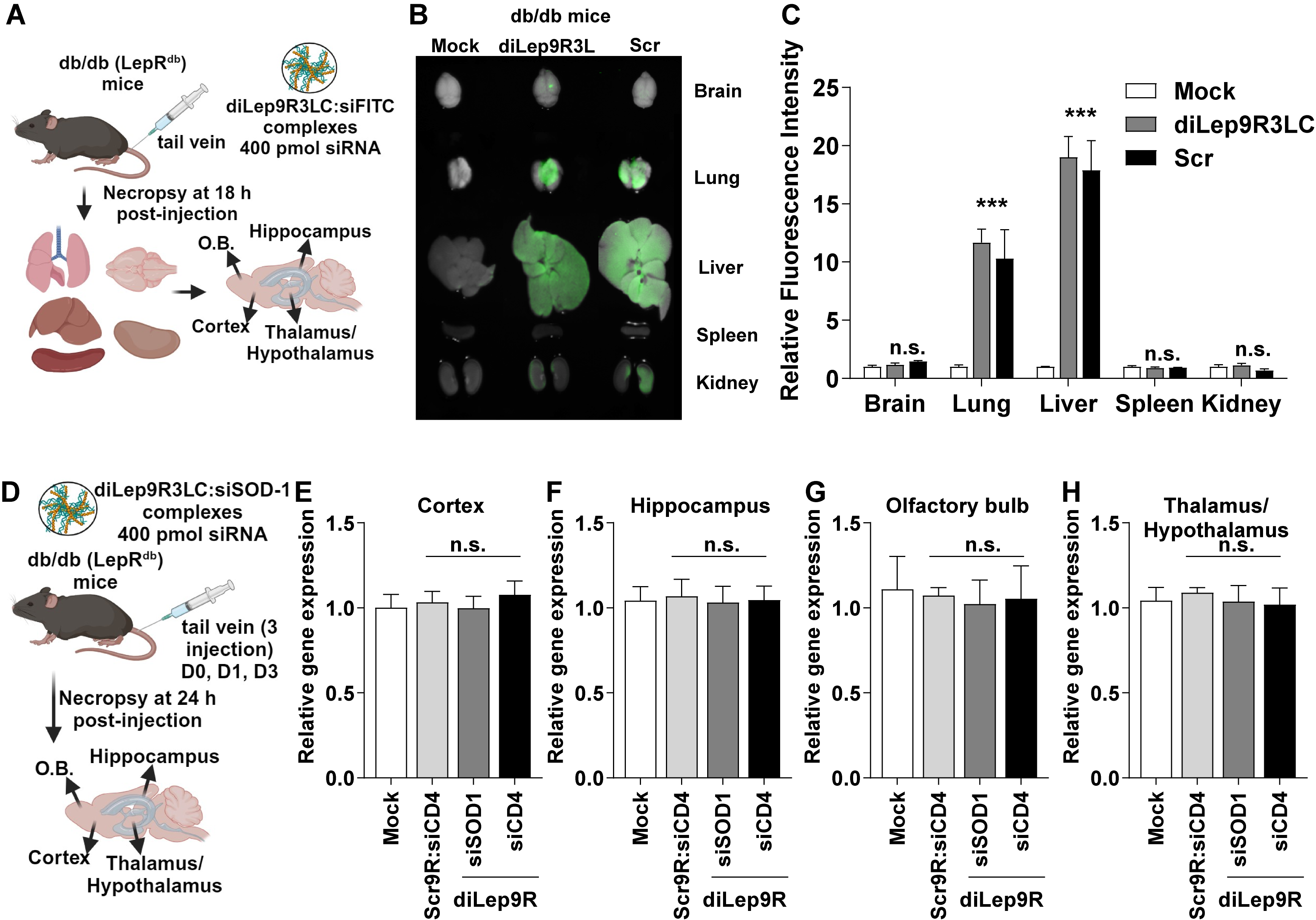

Supplement: Supplementary 1 — Figs. S1 to S5 [file bmr.0027.f1.zip › Fig S3.tif]

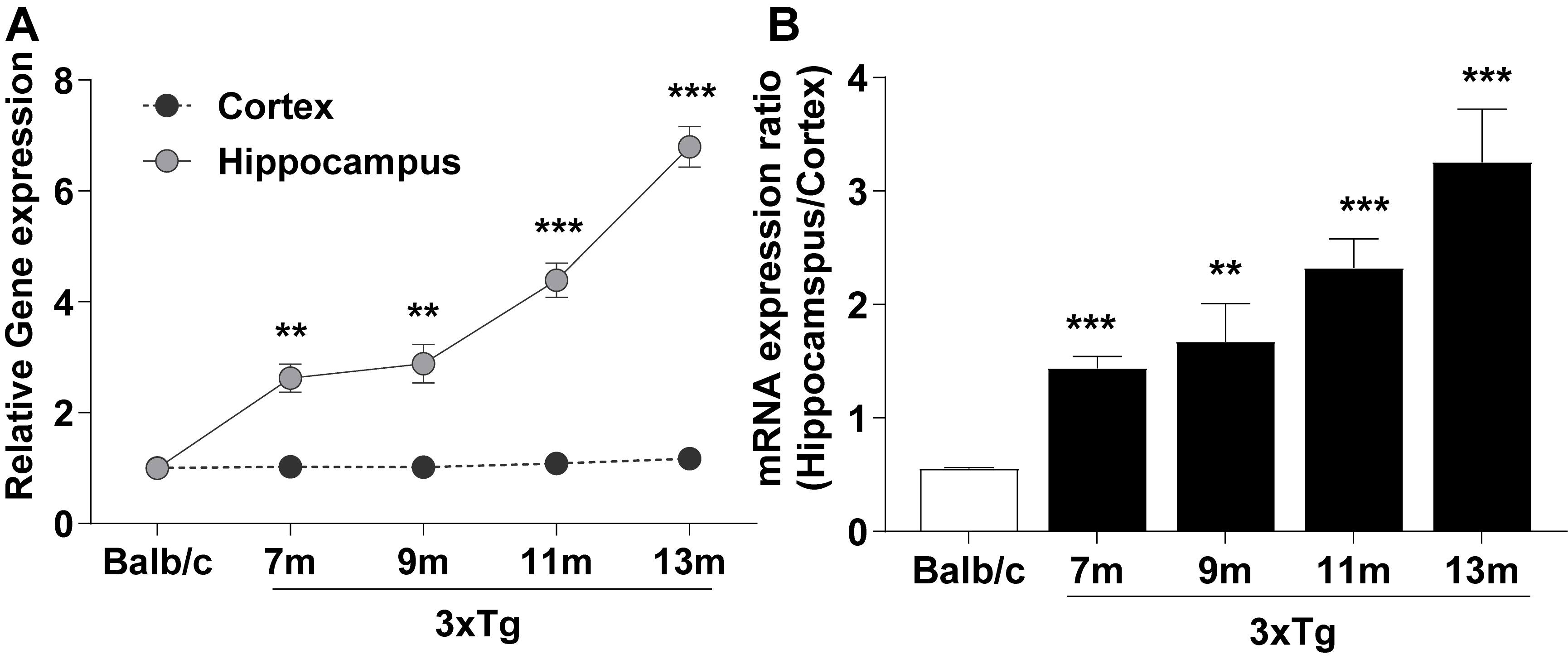

Supplement: Supplementary 1 — Figs. S1 to S5 [file bmr.0027.f1.zip › Fig S4.tif]

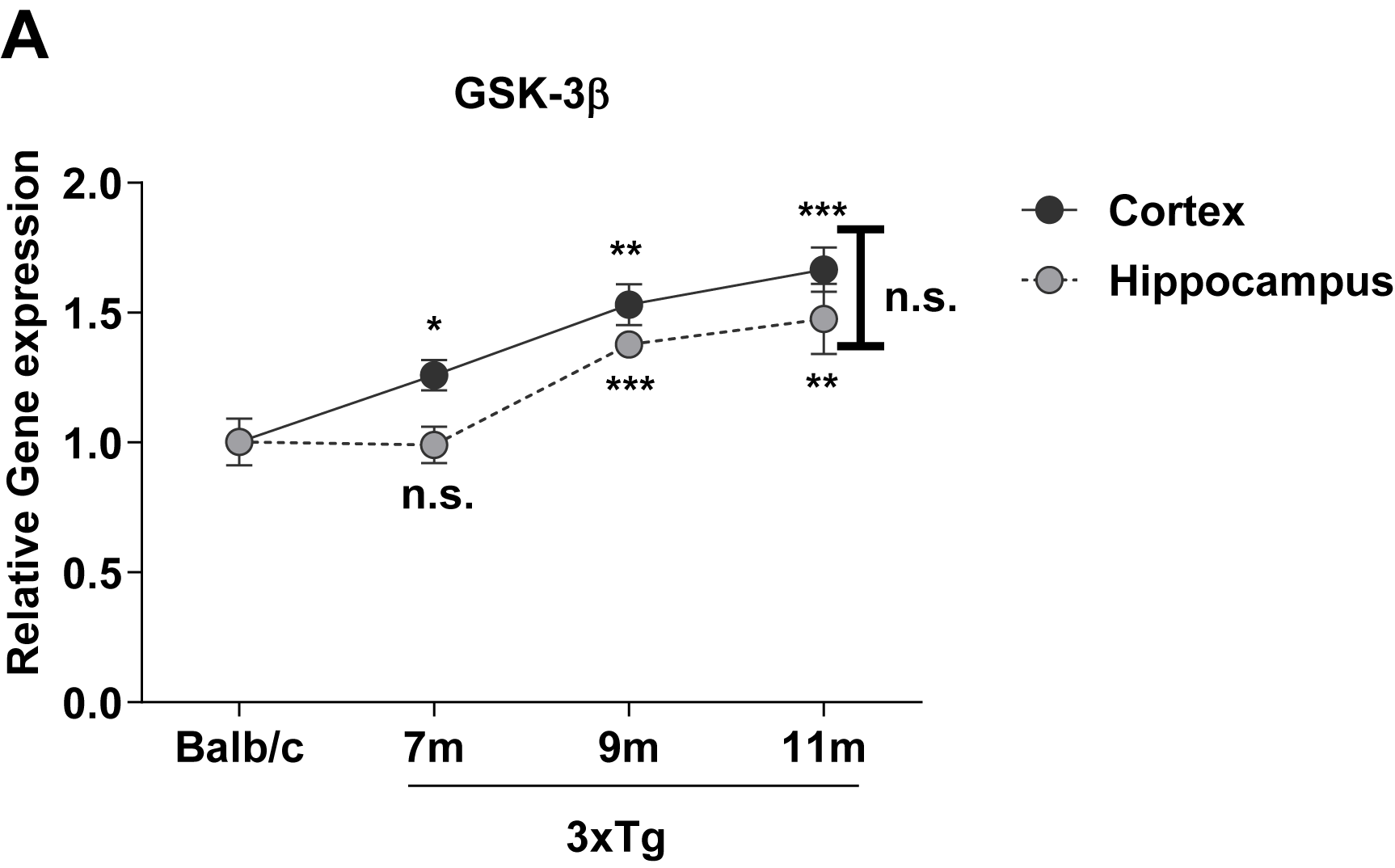

Supplement: Supplementary 1 — Figs. S1 to S5 [file bmr.0027.f1.zip › Fig S5.tif]
